# Health in older age: The German Ageing Survey (DEAS)

**DOI:** 10.1186/1753-6561-7-S4-S11

**Published:** 2013-08-23

**Authors:** Susanne Wurm, Sonja Nowossadeck, Clemens Tesch-Römer

**Affiliations:** 1 German Centre of Gerontology, Berlin, Germany

## 

How healthy are people as they age, what are possible causes and consequences, and what can be derived from this with a view towards health promotion in older people? The German Ageing Survey, funded by the Federal Ministry of Family Affairs, Senior Citizens, Women and Youth (DEAS; http://www.german-ageing-survey.de) provides data to answer these questions. DEAS is an on-going population-based, representative survey of community-dwelling people living in Germany. The survey started in 1996, and the fifth wave will be conducted in 2014. It comprises cohort sequential data, i.e., both repeated cross-sectional surveys and panel data for people aged 40 and over.

To date, DEAS has considered people born between 1911 and 1968, and over 14,700 participants have been interviewed face-to-face and been given written tests and questionnaires. The survey aims at knowing more about the living conditions and the quality of life of people in middle-age and later life, and at knowing more about changes over time, both on the individual and the societal level. Thus DEAS covers a large range of subject areas, including family and social networks, work and retirement, participation, economic situation, well-being, psychosocial resources, as well as health and health behaviour. The data allow both the analysis of individual health trajectories and health changes on the societal level: How healthy will older people be in the next years or decades? For this purpose, different birth cohorts can be compared that have reached the same age at different points in time.

The present findings suggest that birth cohorts born later report less illnesses, lower ailments and higher physical activity, which points to better health and health behaviour of subsequent cohorts. Overall, these findings are in line with those from other surveys in Germany, though the comparison of different studies is limited. One reason for this is the use of different health indicators, which is perhaps most evident in the measurement of chronic illnesses and multi-morbidity. Additionally, other factors such as the in- or exclusion of very old or institutionalised people and the use of cross-sectional surveys versus panel data might contribute to heterogeneous findings on health trends.

For the study of health trends in ageing societies, multiple health indicators are needed to allow for the fact that differences between various health components become more pronounced in later life. Aiming at harmonising concepts and instruments to assess healthy ageing, as initiated by the Robert Koch Institute, is therefore highly appreciated.

